# Flash Flaming Improves Flow Properties of Mediterranean Grasses Used for Direct Seeding

**DOI:** 10.3390/plants9121699

**Published:** 2020-12-03

**Authors:** Bianca Berto, Todd E. Erickson, Alison L. Ritchie

**Affiliations:** 1 School of Biological Sciences, The University of Western Australia, Crawley 6009, Australia; todd.erickson@uwa.edu.au (T.E.E.); alison.ritchie@uwa.edu.au (A.L.R.); 2 Kings Park Science, Department of Biodiversity, Conservation and Attractions, Kings Park 6005, Australia

**Keywords:** flash flaming, direct seeding, seed handling, native grasses, seed enhancement technology, seed cleaning, grassland restoration, germination

## Abstract

The demand for native grasses is increasing in restoration and agriculture, though their use is often limited due to seed handling challenges. The external structures surrounding the grass seed (i.e., the floret) possess hairs, awns, and appendages which create blockages in conventional seeding equipment. Flash flaming is a patented technology which allows precision exposure of floret material to flames to singe off hairs and appendages. We used two grasses native to Mediterranean ecosystems of Western Australia (*Amphipogon turbinatus* R.Br. and *Neurachne alopecuoidea* R.Br.) to evaluate the effects of different flaming techniques on flow properties and germination. Flaming significantly improved flowability in both species and had both neutral (*A. turbinatus*) and negative (*N. alopecuroidea*) effects on germination. Flaming torch size influenced germination, though flaming temperature (low or high) and whether this was kept constant or alternating had no effect. The best evaluation of germination following flaming was achieved by cleaning flamed florets to seed and/or germinating in the presence of karrikinolide (KAR_1_) or gibberellic acid (GA_3_). We suggest that flaming settings (particularly torch size) require species-specific evaluation and optimisation. Removing seeds from flamed florets and germination testing this material in the presence of stimulants may be a useful protocol for future flaming evaluations.

## 1. Introduction

Many Australian native grasses are gaining attention in commercial industries due to increased demand in restoration projects [1] and for their value in agriculture as pasture grasses that are well-adapted to dry climates [2]. The use of native grasses, however, is often limited due to seed handling challenges [3,4]. The outer dispersal unit of grass seeds (known as the floret, consisting of a palea and lemma) are commonly covered in hairs, awns, and appendages which can vary substantially in shape, size, and length [5]. While these appendages commonly play a valuable role in natural dispersal and recruitment, they present a significant challenge for direct-seeding [3,6]. The appendages of intact grass florets commonly cause bridging or blockages in seeding machinery, and highly inconsistent distribution and fluctuations in seeding rates [7,8]. Imprecise seeding can then lead to poor establishment rates, a widely recognised cost and cause of seed wastage in direct-seeding efforts [9].

Numerous methods have been developed to remove appendages or entire floret structures. These include manually rubbing material through ribbed rubber mats, immersion in concentrated sulphuric acid (i.e., acid-digestion), and precision flash flaming [3,4,10]. Of these, flash flaming [10] is the most scalable and safest technique currently being evaluated in Australia [8,11,12]. Flash flaming allows floret material to be rapidly passed through an open flame, gradually singeing off appendages without damaging the seeds encased within the florets [11,13]. While the technology is still under development, it has shown great success in improving seed handling, coating potential [12,13], and even germination in species of *Triodia* (Poaceae) when flamed using commercial-scale flaming machines [11].

To broaden the applicability of this commercial-scale technology and improve our knowledge of the best methods of use, it is important to evaluate the effects flash flaming may have on plant establishment across a range of species using a range of different flaming settings. Many variables of the flaming process can be adjusted, such as flame temperature, distance and angle of the flame relative to the path of florets, rotation plate speed (i.e., how frequently florets are passed through the flame), and the type of flaming torch (different flaming torches produce different flame shapes and sizes). Testing a variety of flaming techniques is therefore warranted to develop our understanding of which variables of the flaming process are most likely to enhance or compromise seed handling and germination.

We trialled various flash flaming treatments on two grasses native to the Mediterranean ecosystems of southwest Western Australia (*Amphipogon turbinatus* R.Br. and *Neurachne alopecuroidea* R.Br). Both species have commercial value as native pasture grasses and biological value in restoration projects [14,15]. The southwest of Western Australia where the study species are distributed has been subject to significant land clearing due to development for agricultural purposes, namely the Wheatbelt [16]. Land management challenges such as decreased rainfall, increased water table level, and dryland salinity throughout southwest Western Australia have raised the need for land reclamation via the preservation and the reintroduction of native vegetation [16,17]. The two study species are well-suited to such projects, though both possess significant awns and hairs which limit their use in direct-seeding efforts. For both species, flash flaming has the potential to reduce or entirely remove the appendages and the hairs which make these species challenging to handle and pass through conventional seeding equipment.

We evaluated the effects of different flash flaming techniques on floret appendage removal, flowability properties, and germination in *A. turbinatus* and *N. alopecuroidea*. Two different torches (small and large) were used at constant or alternating high and low temperatures. Physical changes, bulk density improvements, and flowability properties (i.e., volume, weight and number of florets passed per minute, and number of florets per gram) were analysed for each flaming treatment. Germination tests of flamed floret material with and without the presence of karrikinolide (KAR_1_) and/or gibberellic acid (GA_3_) were also performed. To eliminate additional barriers to germination and promote the uppermost expression of germination, flamed material was also cleaned to seed and tested in the presence of KAR_1_ or GA_3_ as appropriate. It was hypothesised that flash flaming would result in significant improvements in flowability properties compared to untreated florets, and that germination responses would be species-specific and dependent on the different flaming treatments.

## 2. Results

**Figure 1:**
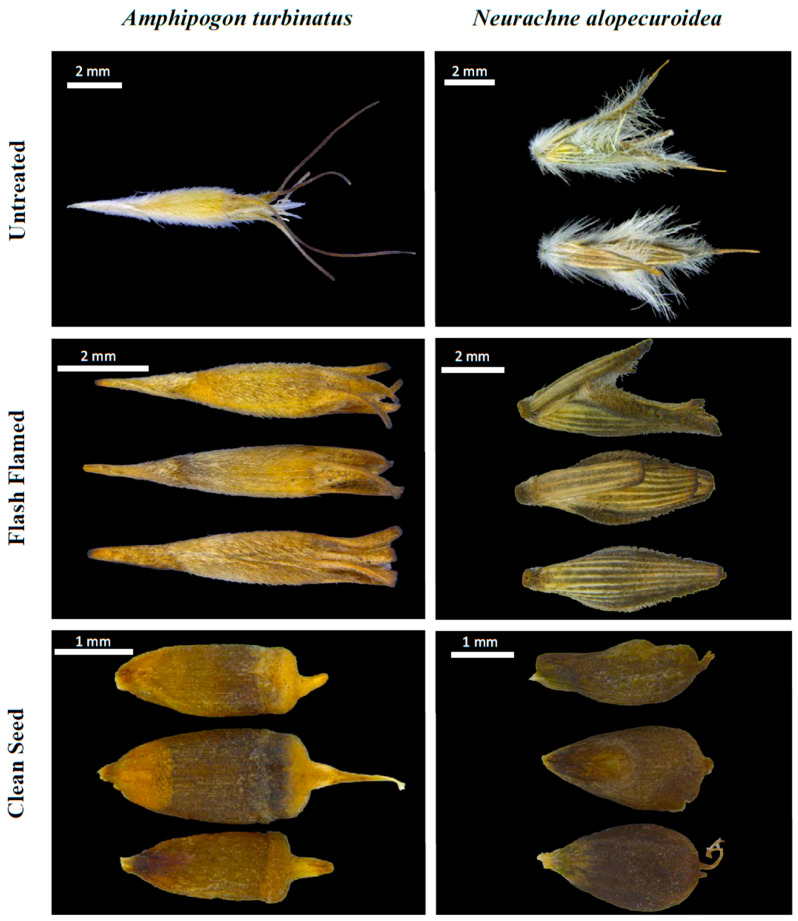
High resolution images of untreated florets (top row), flash flamed florets (middle row), and clean seeds (bottom row) of *Amphipogon turbinatus* (left column) and *Neurachne alopecuroidea* (right column).

Flash flaming successfully reduced awn length in *A. turbinatus* and removed fine hairs and shortened appendages in *N. alopecuriodea* (Figure 1). Bulk density was substantially improved. On average, 1 L samples were reduced to 188 ± 24 mL and 238 ± 13 mL and weight reduced by 30% and 24% for *A. turbinatus* and *N. alopecuroidea*, respectively (see Supplementary Materials, Tables S1 and S2).

**Figure 2:**
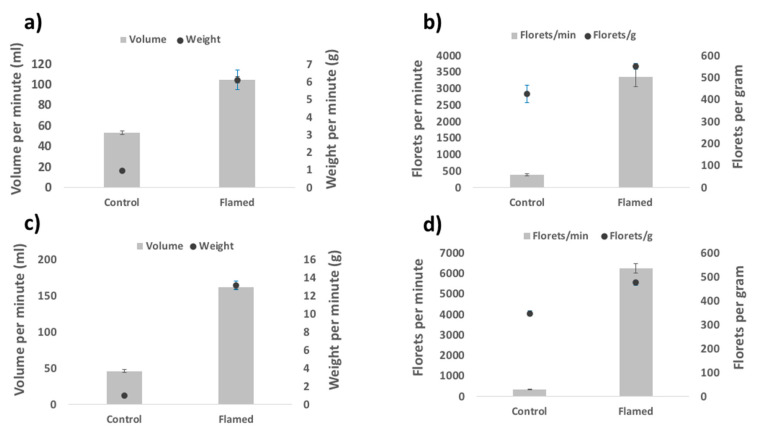
Mean values for flowability properties volume, weight, and florets passed per minute, and the number of florets per gram for *Amphipogon turbinatus* and *Neurchne alopecuroidea* (panels **a**,**b** and **c**,**d**, respectively). Comparisons are displayed for treatment groups control and flamed. Differences in flowability properties between the different flaming treatments were generally insignificant (Table S7), thus flaming data were pooled for the purposes of graphical representation (pooled values are provided in Table S6). Error bars represent standard error of the mean.

These physical and bulk density changes were also associated with significant flowability improvements following flaming. Volume, weight, and number of florets that could be passed through the mechanised seeding box per minute increased significantly for flamed (pooled data) versus non-flamed florets for both species (*p* < 0.001; Table 1, Figure 2; Table S6). The number of florets per gram of floret material also improved significantly for both species following flaming (*p* < 0.01; Table 1, Tables S5 and S6). Differences in the flowability properties between different flaming treatments were generally insignificant, with no single treatment consistently performing better (or worse) than others across all flowability properties (Table S7). Only six out of twenty-four pairwise comparisons produced significant differences between flaming treatments for *A. turbinatus*, while no significant differences were found between flaming treatments in *N. alopecuroidea* (Table S7).

**Figure 3:**
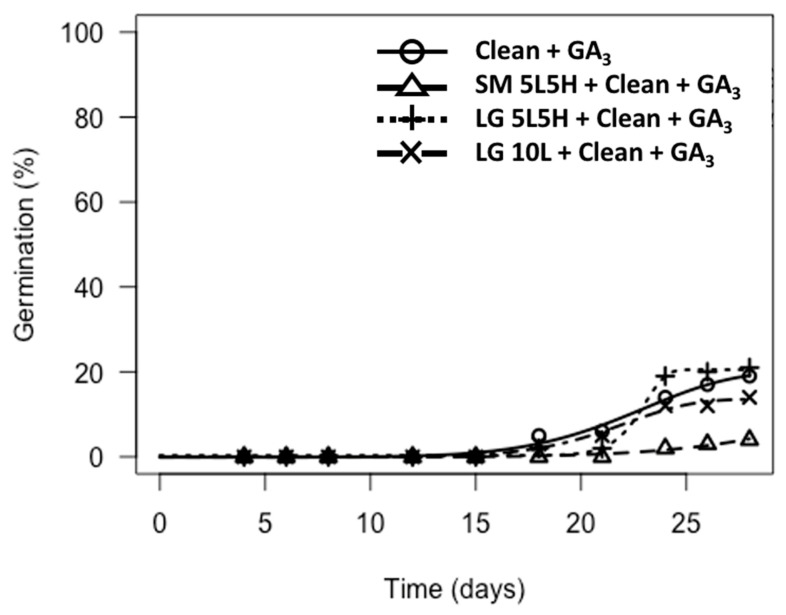
Germination of *A. turbinatus* over time for treatments that included cleaning to seed and testing on GA_3_-agar (“Clean + GA_3_”) and where germination was present. The following abbreviations are used: Clean = cleaned to seed, GA_3_ = tested on GA_3_-agar, SM = small torch, LG = large torch, 5L5H = exposed to a low flaming temperature for 5 min followed by a high flaming temperature for 5 min, 10L = exposed to a constant low flaming temperature for 10 min. Note that treatments that did not include “Clean + GA_3_” did not produce any germination, nor did the treatment SM 10L + Clean + GA_3_, and are therefore not represented in figures.

Germination was only observed for *A. turbinatus* when cleaned to seeds (flamed or not flamed) and tested on GA_3_-agar (Figure 3; Tables S8 and S9). Clean seeds and florets (flamed or not flamed) tested on water-agar or KAR_1_-agar failed to germinate, as did florets tested on GA_3_-agar (Table S9).

Flamed *A. turbinatus* florets (using the large torch) that were cleaned to seeds and tested on GA_3_-agar showed low germination (<25%), with only small differences (<10%) being observed between the constant versus alternating flame temperature treatments (Tables S9 and S10). Florets exposed to these treatments also had similar germination to clean seeds (non-flamed) tested on GA_3_-agar (15–20%, Figure 3; Table S11). *T*_50m_ of all treated seeds of *A. turbinatus* with >5% germination was approximately 23 d (Figure 3). Florets that were flamed using the small torch and then cleaned to seeds and tested on GA_3_-agar produced low (<5%) germination, though germination was not significantly lower than florets treated with the large torch and then cleaned to seeds and tested on GA_3_-agar (*p* > 0.05, Figure 3; Table S10).

**Figure 4:**
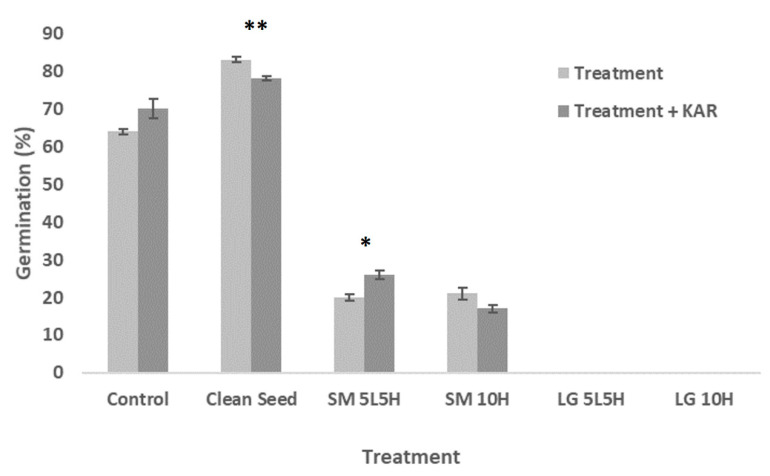
Maximum germination achieved for *N. alopecuroidea* for each treatment (flaming or seed cleaning) when tested on water-agar (“Treatment”) compared to each treatment when tested on KAR_1_-agar (“Treatment + KAR”). Error bars represent standard error of the mean. Asterisks “*” and “**” denote significance levels 0.05 and 0.01, respectively, and indicate a significant difference between the treatment and treatment + KAR_1_.

Germination of untreated *N. alopecuroidea* florets was lower (~64%) when compared to clean seeds (~83%) (*p* < 0.001, Figure 4; Table S12). Florets flamed with the small torch had low germination (20–25%) compared to the control group (*p* < 0.001), and germination was absent in florets flamed with the large torch (Figure 4; Table S12). Significant responses to KAR_1_ exposure were observed for clean seed (negative response, *p* < 0.01) and the alternating small torch (SM 5L5H) treatment only (positive response, *p* < 0.05) (Figure 4; Table S13).

**Figure 5:**
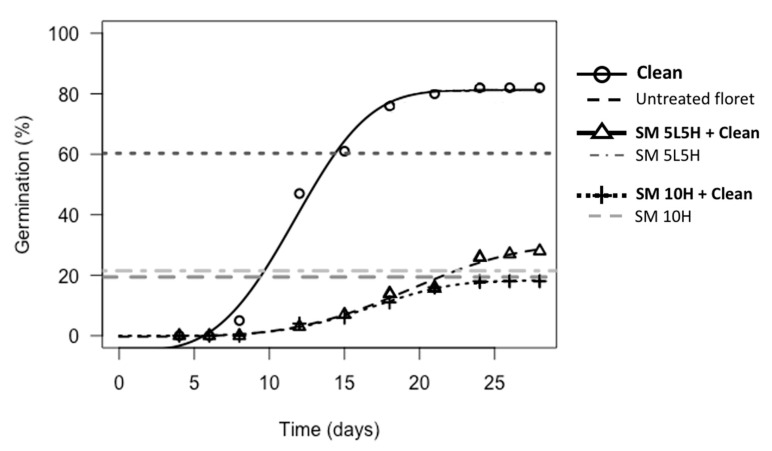
Germination of *N. alopecuroidea* untreated florets and florets flamed with the small torch with and without subsequent seed cleaning. Treatments that included seed cleaning are represented with dose-response curves, while those that did not include seed cleaning are represented by horizontal dashed lines (maximum germination at 45 d) as a comparison point. The following abbreviations are used: Clean = cleaned to seed, SM = small torch, 5L5H = exposed to a low flaming temperature for 5 min followed by a high flaming temperature for 5 min, 10H = exposed to a constant high flaming temperature for 10 min.

*Neurachne alopecuroidea* florets that were treated with a small flame and then cleaned to seeds resulted in improved germination (5–10%) compared to the same flaming treatments without subsequent seed cleaning (Table S13). Of these comparisons, florets treated with the small torch alternating temperature treatment (SM 5L5H + Clean) showed a significant improvement compared to the same treatment without subsequent cleaning (11% improvement, *p* < 0.05; Table S13). Germination was still lower (*p* < 0.01) and *T*_50m_ slower for flaming followed by seed cleaning treatments compared to untreated florets cleaned to seeds (Figure 5; Table S14).

## 3. Discussion

Several studies have now shown the advantages of flash flaming to improve the shape and the handling of floret material possessing hairs and appendages that otherwise cause entanglement and blockage in conventional seeding equipment [1,8,10,11,12]. The flaming evaluations conducted in this study demonstrate significant seed handling (flowability) and bulk density improvements in southwest Western Australian native grasses *A. turbinatus* and *N. alopecuroidea*. This study also demonstrates how different flash flaming settings and techniques can significantly impact germination. Methods to promote the uppermost expression of germination following flash flaming (i.e., post-flaming seed cleaning and exposure to germination stimulants) were also explored and may provide a valuable protocol where deeper evaluations of the effects of flash flaming on germination are required. Using contrasting flaming settings and techniques, we observed both neutral and negative effects of flash flaming on germination, with flaming variables (especially torch size) having a significant influence on germination. These outcomes have implications regarding the underlying biology and ecology of the study species and future directions for research and application of the flash flaming technology.

### 3.1. Handling Improvements

Flash flaming significantly improved flowability properties for the two study species, with a significantly greater volume, weight, and number of florets being passed through the seeding machine per minute. The number of florets per gram also increased, as did bulk density. Such improvements demonstrate that flash flaming has the potential to improve the rate and the precision of seeding for species possessing significant hairs and appendages. This is critical in the context of broad-scale restoration efforts where large quantities of seed need to be delivered to sites over short time frames via direct seeding. Furthermore, seeding depth and density are known to significantly influence plant establishment outcomes, and it is therefore critical to optimise these factors [18,19,20]. Improved flowability properties could enable more control over precision seeding, improving plant establishment outcomes and reducing seed waste [1,9,18,21].

The flowability data in this study indicate that different flaming techniques tend to result in similar flowability improvements, though flash flaming settings may still need to be adjusted to accommodate species-specific characteristics. For instance, as observed in this study, lower intensity flaming settings are better suited to removing fine hairs and appendages, while higher flaming intensities are better suited to more coarse bristles and appendages. Florets commonly possess a combination of both fine and coarse hairs, awns, and appendages [5]; therefore, exposing floret material to a high intensity flaming setting immediately may result in combustion. In this study, it was necessary to first apply a low intensity flame to *A. turbinatus* florets to remove finer hairs and appendages before a higher flaming intensity could be applied to remove the remaining coarse appendages.

One limitation of flash flaming observed in this study, however, is that, although the technology is well-suited to removing fine to moderately coarse hairs, bristles, awns, and appendages, it is unable to remove thickened appendages such as the prominent lemma apex in *N. alopecuroidea*. In instances where flaming has been applied at intensities which achieve complete removal of the palea and the lemma, the technology can cause damage to the seed [1]. Consequently, removal of these structures via flaming should be avoided. Interestingly, in the case of *N. alopecuroidea,* flow properties were still improved by up to 19-fold despite flaming being unable to remove the prominent lemma apex. It has previously been observed that the fine hairs and bristles of grass florets are the most significant challenge to seed handling [7]. Therefore, it may not always be necessary to remove thickened appendages, rather focusing on those which are finer to improve flowability (to which flash flaming is well suited).

Finally, improved flowability increases the compatibility of chaffy species with conventional seeding equipment, eliminating the need to modify machinery. Technologies that improve flow rate and seeding consistency have been shown to reduce seeding time and labour, overall mitigating the costs of implementing the technology [21]. While a cost–benefit analysis has not yet been completed to assess flash flaming at a broad scale, the technology is relatively cheap to implement (e.g., main overhead costs are labour, and minor costs of liquefied petroleum gas) and may be more successful in achieving uniform seeding rates than costly modifications to seeding equipment. For instance, a number of modifications to seeding equipment have been developed with seed drills being most widely implemented [18]. However, it has been acknowledged that seed drills cannot achieve uniform seeding rates, and seeding depth is often imprecise [3,20]. Flaming therefore presents a viable and relatively inexpensive alternative to modifying seeding equipment [11].

### 3.2. Germination Responses

Flash flaming has been found to both enhance [13] and compromise [1] germination capacity. This study demonstrated neutral (*A. turbinatus*) and negative (*N. alopecuroidea*) germination responses to flash flaming. Critically, selecting appropriate flaming settings was imperative to germination, with optimal settings being species-specific. For instance, *A. turbinatus* germination responses were better when the large flaming torch was used, while *N. alopecuroidea* germination capacity was lost entirely using the large torch but retained (though reduced) when using the small flaming torch. High and low temperature variations were tested, and holding these constant or alternating did not appear to influence germination in this study.

One way that flash flaming has been suggested to positively influence germination is via weakening of floret structures which, prior to flaming, create mechanical impedances to embryo growth and expansion [8,13]. Both species in this study demonstrated increased germination once cleaned to seed, suggesting floret-imposed dormancy is a significant constraint to germination. Despite this, flaming was unable to overcome dormancy mechanisms in *A. turbinatus* as cleaning to seed, and exposure to GA_3_ was still necessary before germination was observed. For *N. alopecuroidea*, the negative effects of flaming (e.g., exposure to potentially damaging temperatures) outweighed any possible benefits of alleviating mechanical impedances imposed by floret structures.

Excessive intensity and duration of flame exposure during flash flaming has previously been shown to be detrimental to germination capacity [1]. Individual species and seeds within the same species have different lethal temperature thresholds owing to factors such as seed size and seed moisture content [22]. It might be expected that smaller seeded species with minimal investment in external structures have lower temperature thresholds than larger seeded species with reinforced external structures, meaning the former are less suited to flaming or require lower intensity flaming settings.

The two species considered in this study are from Mediterranean ecosystems where wildfires are an important ecological process [23]. During wildfires, the temperatures that seeds can be exposed to vary considerably depending on the soil depth seeds are located at, the site characteristics, and the fire intensity [22,24]. At the soil surface, temperatures commonly exceed 160 °C during wildfires (the upper temperature recorded during our flaming treatments), with temperatures as high as 400–800 °C being recorded at the soil surface during grass fires [25]. These temperatures are rarely maintained for longer than 10 min [24,25,26]. With increasing soil depth, the temperatures experienced during fire decrease significantly (often <60 °C at depths of 2 cm or more), though the heat is retained for longer [24,25,26]. The conditions produced by flash flaming correspond with temperatures most likely to be experienced at the soil surface or immediately below (<1 cm) [26]. The flame exposure durations used in this study, however, may be on the upper limit of what would typically be experienced in nature. Managing flaming settings to ensure seeds only experience conditions akin to those experienced during wildfire could allow germination capacity to be retained following flaming treatments, though this requires further research. Additionally, taking into account lethal temperature thresholds of seeds and the traits which influence this threshold (e.g., embryo type) [22,27] may help to inform decisions around flaming settings. For instance, the use of cooling or rest periods, smaller flame-sizes, and shorter flaming durations may be beneficial for species with lower temperature thresholds such as *N. alopecuroidea*.

In species where revised flaming methods continue to be detrimental to germination capacity, alternative methods of appendage removal such as acid digestion warrant exploration. Acid digestion involves the immersion of florets in sulfuric acid (H_2_SO_4_), leading to the digestion of fine hairs and appendages [4]. This method has already proven to be effective while also maintaining or improving germination capacity in a number of valuable native grasses such as *Austrostipa scabra*, *Chloris truncata*, *Rytidosperma caespitosum*, *Rytidosperma geniculatum*, and *Microlaena stipoides* [1,4], though it has not yet been upscaled and is likely to be more resource intensive than flaming.

Although the germination responses to flaming treatments in this study were neutral and negative, these findings are highly relevant to the development of the flash flaming technology and application by practitioners. Poor and unpredictable supply of native grass seed is a common shortfall for research and restoration. The price of native grass seed reflects this challenge, with one of Australia’s largest native seed merchants (Nindethana Seed Service Pty Ltd., King River, Australia) advertising *A. turbinatus* and *N. alopecuroidea* for AUD $10,000/kg and $6000/kg, respectively, at the time of writing [28]. These factors considered, it is critical to highlight both successful and unsuccessful applications of seed technologies such as flash flaming to guide practitioner decisions as well as future research directions. Furthermore, the differences seen in germination responses when flaming variables were adjusted (especially torch size) emphasise how a potentially useful technology can become detrimental under the incorrect settings (e.g., complete loss of germination capacity using a large flaming torch versus reduced germination when using a small flaming torch in *N. alopecuroidea*).

The contrasting germination responses to flash flaming across studies raises the question of how to assess candidates suited to flaming. This may be achieved by considering the underlying ecology and adaptations of the species to other fire-related cues. In this study, both species were non-responsive to the smoke-derived compound KAR_1_, possibly suggesting that they are not adapted to respond to fire-related cues. This may explain the neutral or the negative germination outcomes observed in response to flaming. In comparison, arid zone species which are highly smoke-responsive such as *Triodia wiseana* have shown germination improvements in response to flash flaming [13]. Therefore, species which are responsive to fire-related cues such as KAR_1_ exposure might be expected to be good candidates for flash flaming, though this relationship requires further exploration.

### 3.3. Flaming Evaluation Techniques

Finally, this study also trialled a novel approach in evaluating the impacts of flaming on germination beyond what has previously been tested (e.g., on water only [1]). Cleaning to seed and treating with an appropriate dormancy break treatment is a common method for enhancing germination expression in grasses [29,30]. By cleaning to seed florets which had previously been flamed and testing these seeds on a growth medium inoculated with a dormancy break or germination stimulating chemical (i.e., KAR_1_ or GA_3_), it was possible to enhance germination expression for each species following flaming treatments [13,29]. An alternative to this may be to perform seed priming, which may include inoculation with dormancy break chemicals following flaming, as this has also been shown to further enhance germination in flamed florets [12].

Even under the best treatment (i.e., seed cleaning and GA_3_ exposure), germination remained low (<25%) for *A. turbinatus*, suggesting that dormancy-break had not been achieved in the majority of the seed batch, or the seed batch was partly non-viable. In instances where the majority of the seed batch remains dormant, it is challenging to accurately assess the effects of flaming on germination. For species such as *A. turbinatus*, where dormancy cannot be alleviated using common methods (seed cleaning, KAR_1_ exposure, GA_3_ exposure), it may be necessary to provide alternative dormancy break treatments (e.g., after-ripening, cold stratification) to more accurately evaluate the impacts of flaming on germination [29]. For *N. alopecuroidea*, germinating flamed florets that were then cleaned to seed improved germination slightly compared to germinating flamed florets left intact, though germination remained clearly compromised. This provided sound evidence that the flaming treatments applied in this study were detrimental to the germination capacity of *N. alopecuroidea*.

We suggest that comparing flamed material left intact with flamed material that is cleaned to seed and testing germination in the presence of appropriate stimulants (or following other suitable dormancy-break treatments) may be a used as a protocol for assessing the detailed effects of flash flaming on the germination capacity of targeted species. This may be a valuable tool where the initial germination capacity of a seed batch is low or where strong dormancy mechanisms persist and obscure the true effects of flaming on germination.

## 4. Materials and Methods

### 4.1. Study Species

**Figure 6:**
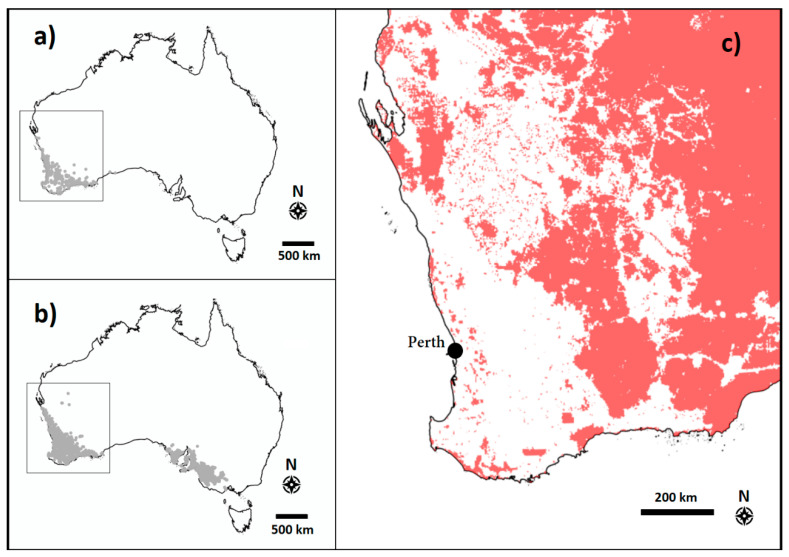
Distribution maps for *Amphipogon turbinatus* (**a**) and *Neurachne alopecuroidea* (**b**), and the extent of unaltered native vegetation in southwest Western Australia (**c**). The boxes in panels **a** and **b** indicate the area enlarged in panel **c**. Maps generated from Atlas of Living Australia using species occurrence records data (**a** and **b**) and selecting “remaining vegetation” within the “Vegetation–Condition” layer (**c**) [32,33,34].

*Amphipogon turbinatus* is distributed across the southwest of Western Australia (Figure 6a), where mean annual rainfall and mean temperatures are 400–1500 mm and 12–24 °C, respectively [31]. The florets of *A. turbinatus* have five straight, apical awns of up to 12.5 mm [5] which cause florets to become entangled in one another.

*Neurachne alopecuroidea* is widely distributed across temperate and semi-arid zones of southern Australia (Figure 6b), where mean annual rainfall and mean temperatures are 300–1500 mm and 12–24 °C, respectively [31]. Florets have a distinct divide between the palea and the lemma, prolific hairs resulting in florets “adhering” to one another, and poor bulk density.

Where the two study species occur within Western Australia, land clearing for agricultural development (namely the Wheatbelt) has resulted in extensive loss of intact native vegetation throughout southwest Western Australia (Figure 6c) [16].

### 4.2. Flaming Treatments

Flaming treatments were performed at the University of Western Australia using a commercial-scale flaming apparatus (described in [8,11]; see Supplementary Materials; Figure S1). Four different flaming variations were tested on each species, with each flaming treatment lasting 10 min (Table 2). The main variables altered were the type of flaming torch (“small” or “large”; sensu [11]), flame temperature (“low” or “high”) and whether the flame temperature was held constant (i.e., same temperature for 10 min) or alternated (i.e., 5 min “low” followed by 5 min “high” temperature). Low and high flame temperatures were species-specific and determined during trial runs using a heat gun to monitor flame temperature (see Supplementary Materials).

Note that, for *A. turbinatus*, the presence of finer hairs resulted in a higher combustion risk (as also reported for *R. geniculatum* [1]), and it was therefore not possible to conduct a 10 min high temperature treatment. Instead, a 10 min “low” temperature treatment was performed. For *N. alopecuroidea*, the risk of combustion was lower, and the 10 min “high” temperature treatments were preferred.

### 4.3. Bulk Density and Flowability Measurements

The volume and the weight of floret material were recorded before and after flaming to determine improvements in bulk density (Tables S1 and S2). Flowability properties for each species were determined using a custom-built mechanised seeding box with a fluted roller (AUSBOX^TM^; Figure S2). The seed box was divided to create a small 4 × 3 cm aperture. Three replicates of 50 mL samples per treatment were passed through the 4 × 3 cm aperture with the fluted roller set to 10 rpm for all runs. The time taken to pass each 50 mL sample was recorded (Table S3). To determine the weight and the number of florets being passed per minute, a total of three 5 mL samples for each treatment were weighed (to provide weight per mL), and the total number of florets were counted (to provide the total number of florets per mL) (Table S4).

### 4.4. Germination Testing

Following flaming treatments, floret fill was improved to 100% using a combination of aspiration (“Zig Zag” Selecta, Machinefabriek BV, Enkhuizenm The Netherlands) to sort samples into a heavy (i.e., florets containing a seed) and light (i.e., florets not containing a seed) fraction, followed by X-ray analysis (Faxitron MX-20 digital X-ray cabinet, Tucson, AZ, USA) to allow identification and manual removal of any remaining empty florets within the heavy fraction.

Prior to germination testing, florets and seeds were sterilised in a 2% (*w*/*v*) calcium hypochlorite (Ca(OCl)_2_) solution. Untreated florets (control), clean seeds (non-flamed), flamed florets, and flamed florets cleaned to seeds were tested on up to three different 0.7% (*w*/*v*) agar growth mediums: (1) water-agar, 2) agar containing a 0.67 μm concentration karrikinolide solution (KAR_1_; 3-methyl-2H-furo [2–c]pyran-2-one (synthesized following the methods of [35]), which is referred to as “KAR_1_-agar” hereafter, and 3) agar with a 100 ppm concentration of ProGibb^®^ gibberellic acid (Sumitomo Chemical, Epping, NSW, Australia), which is referred to as “GA_3_-agar” hereafter. For treatments where florets were cleaned to seeds, florets were gently rubbed between ribbed rubber mats to remove the external structures. A total of four replicates (90 mm Petri dishes) each containing 25 filled florets were produced for each treatment. All germination tests were conducted at a constant temperature of 15 °C under a 12 h light/dark cycle for 28 days. In instances where germination rate had not decreased by day 28, germination tests were extended to 45 days (see Supplementary Materials).

Two rounds of germination testing (commencing on different dates) were conducted to optimize the method (or protocol) of assessing flaming effects. In the first round, flamed florets, clean seeds, and untreated florets were tested on both water- and KAR_1_-agar (Table 3). In the second round, flamed florets were cleaned to seeds for optimal flaming treatments in *N. alopecuroidea* and for all treatments for *A. turbinatus* (Table 3). The clean seeds produced from flamed florets were then tested on water-agar (if no response to KAR_1_ was observed) or GA_3_-agar (if germination from all previous tests, including exposure to KAR_1_, were poor) (Table 3).

### 4.5. Data Analysis

Summary statistics such as total weight and volume of florets passed per minute and number of florets per ml and per gram were calculated for the flowability data. Due to the low number of replicates (*n* = 3) and variance being heterogeneous for some (but not all) comparisons, individual *t*-tests were performed in Excel to compare flowability properties of flamed against non-flamed material. Where variance was equal between groups, a Student’s *t*-test was used, while Welch’s *t*-test was used where variance was heterogeneous (Tables S5–S7).

All germination data were analysed in R (R Core Team 2019) using the dose-response curve (*drc*) package [36] and Kruskal–Wallis non-parametric one-way ANOVA (as assumptions of ANOVA were violated) with Dunn’s post-hoc multiple-comparisons (all parameter estimates and test statistics can be found in Tables S8–S14). Dose response models were fitted to germination over time data following the assumptions of the four-parameter Weibull function [36]. Maximum germination and time to 50% of the maximum germination (*T*_50m_; [37]), parameters “*d*” and “*e*”, respectively, were considered in comparisons. Separate controls were run for each of the two rounds of germination testing, as these were conducted over two separate dates. The separate controls were compared to ensure no statistically significant differences were present before comparing treatments performed on separate dates. Statistical comparisons were only made for maximum germination and not *T*_50m_ to avoid making misleading comparisons in time to 50% maximum germination (*T*_50m_) where maximum germination between treatments differs [37].

Inaccurate parameter estimates can be generated from the dose-response model for germination data if (1) germination for a treatment is low (1–5%) or (2) germination rate has not decreased by the end of the experiment (day 28), failing to create a sigmoidal curve when plotted. To account for these scenarios, treatments which produced low germination outcomes (1–5%) were instead analysed using Kruskal–Wallis non-parametric one-way ANOVA (as assumptions of ANOVA were violated) and Dunn’s post-hoc multiple-comparisons to compare mean maximum germination only (estimates for *T*_50m_ could not be provided). In instances where germination had not plateaued by day 28, germination was recorded for a longer period (45 d) until a decrease in germination rate was observed, and these additional data were used to predict parameter estimates using dose-response modelling. The analysis method used for each treatment is specified in the Supplementary Materials.

## Figures and Tables

**Table 1:** Flowability data showing the fold-increase in volume per minute, weight per minute, and number of florets per minute passed through the custom-built mechanised seeder compared to the control. The number of florets per gram is also provided. Asterisks “*”, “**”, and “***” represent significance levels 0.05, 0.01, 0.001, respectively. For example, “(1.8**)” indicates that the treatment resulted in a 1.8-fold improvement on the control and that this increase was significant (*p* < 0.01). The mean ± standard error values for the control have been provided.

Species	Treatment	Vol/min	Weight/min	Florets/min	Florets/g
*A. turbinatus*	Control	53 ± 1.73	0.93 ± 0.001	392 ± 33	423 ± 39
	SM 10L	1.8 **	6.0 **	8.2 **	1.4 *
	SM 5L5H	1.9 *	9.7 **	12.2 *	1.2
	LG 10L	2.2 **	4.7 **	6.1 **	1.3
	LG 5L5H	2.0 ***	5.8 **	7.6 **	1.3 **
	Flamed (pooled)	2.0 ***	6.6 ***	8.5 ***	1.3 **
*N. alopecuroidea*	Control	46 ± 2.34	0.96 ± 0.001	331 ± 15	347 ± 10
	SM 10H	3.6 ***	14.6 ***	20.7 **	1.4 **
	SM 5L5H	3.5 **	12.4 **	17.7 **	1.4 **
	LG 10H	3.5 **	14.4 **	18.6 *	1.3
	LG 5L5H	3.5 **	13.5 *	18.2 **	1.4 **
	Flamed (pooled)	3.5 ***	13.7 ***	18.8 ***	1.4 ***

**Table 2:** A summarised description of the four different flaming treatments performed for each *Amphipogon turbinatus* and *Neurachne alopecuroidea*.

Species	Treatment ID	Description
*A. turbinatus*	SM 10L	Small torch 10 min constant low (~150 °C)
	SM 5L5H	Small torch 5 min low (~150 °C) followed by 5 min high (~160 °C)
	LG 10L	Large torch 10 min constant low (~130 °C)
	LG 5L5H	Large torch 5 min low (~130 °C) followed by 5 min high (~140 °C)
*N. alopecuroidea*	SM 10H	Small torch 10 min constant high (~150 °C)
	SM 5L5H	Small torch 5 min low (~150 °C) followed by 5 min high (~160 °C)
	LG 10H	Large torch 10 min constant high (~140 °C)
	LG 5L5H	Large torch 5 min low (~140 °C) followed by 5 min high (~150 °C)

**Table 3:** A list of all treatments tested for each species and the growth mediums which these were tested on. The treatments tested for each species are marked as “1” (tested in round one) and/or “2” (tested in round two). Water, KAR_1_, and GA_3_ indicate whether the treatment was tested on water-agar, KAR_1_-agar, or GA_3_-agar.

*Amphipogon turbinatus*				*Neurachne alopecuroidea*			
Treatment	Water	KAR_1_	GA_3_	Treatment	Water	KAR_1_	GA_3_
Untreated floret (control)	1	1	2	Untreated floret (control)	1	1	
Clean seed	1	1	2	Clean seed	1, 2	1	
SM 10L	1	1	2	SM 10H	1	1	
SM 5L5H	1	1	2	SM 5L5H	1	1	
LG 10L	1	1	2	LG 10H	1	1	
LG 5L5H	1	1	2	LG 5L5H	1	1	
SM 10L + Clean			2	SM 10H + Clean	2		
SM 5L5H + Clean			2	SM 5L5H + Clean	2		
LG 10L + Clean			2	LG 10H + Clean			
LG 5L5H + Clean			2	LG 5L5H + Clean			

## Supplementary Materials

The following are available online at https://www.mdpi.com/2223-7747/9/12/1699/s1, Figure S1. Images of flash flaming apparatus. Figure S2. Images of custom-build seeding machine. Table S1. *Amphipogon turbinatus* volume and weight changes for each flaming treatment. Table S2. *Neurachne alopecuroidea* volume and weight changes for each flaming treatment. Table S3. Time taken to pass 50 mL samples of floret material through mechanised seeder. Table S4. Weight and number of florets recorded for 5 mL samples of each treatment for each species. Table S5. Statistical comparisons for flowability variables for each flaming treatment versus the control (non-flamed) in each species. Table S6. Statistical comparisons for flowability variables for all flaming treatments and pooled flaming data versus control (non-flamed) data in each species. Table S7. Statistical comparisons for flowability variables between the different flaming treatments in each species. Table S8. List of treatments where dose-response model (*drm*) analysis could be used. Table S9. Parameter estimates for maximum germination and *T*_50m_ for *Amphipogon turbinatus*. Table S10. Statistical comparisons for germination across different flaming treatments for *Amphipogon turbinatus*. Table S11. Statistical comparisons for germination of flaming treatments versus their non-flamed equivalent for *Amphipogon turbinatus*. Table S12. Parameter estimates for maximum germination and *T*_50m_ for *Neurachne alopecuroidea*. Table S13. Statistical comparisons for germination across different flaming treatments for *Neurachne alopecuroidea*. Table S14. Statistical comparisons for germination of flaming treatments versus their non-flamed equivalent for *Neurachne alopecuroidea*.
