# Supplementary material for: Flash Flaming Improves Flow Properties of Mediterranean Grasses Used for Direct Seeding
Source: Plants (Basel). 2020 Dec 3;9(12):1699. doi: 10.3390/plants9121699 (PMC7761630; doi:10.3390/plants9121699)
Supplement: Supplementary file 1 [file plants-09-01699-s001.pdf]

Supplementary Materials (This document)

Flash flaming set-up and treatment application

Flowability and bulk density analysis

Statistical analyses and comparisons

Supplementary Tables (S1 to S14) and Figures (S1 to S2)

Flash flaming set-up and treatment application

Preliminary trial runs each lasting 15 mins were conducted to determine the “high” and “low” flaming temperatures for each species and each flaming torch. The low flaming temperatures were determined as being the lowest temperature at which flaming still had an effect on reducing hairs and appendages, while the high flaming temperature was determined as that being the highest temperature that could be applied without risking combustion. The heat was adjusted simply by increasing or decreasing the flow of gas. A heat gun was used to monitor the flame temperature at regular intervals (approx. every 2 mins) to ensure consistency throughout and between treatments. In addition to variables such as the type of torch and the temperature of the flame, the seed flamer has the option of different torch positions (i.e. distance and angle to the stream of seeds), different plate rotation speeds (i.e. the rpm of seeds within the drum), and one or two flaming torches (Fig. S1). For all flaming treatments in this study, a single flaming torch was used and positioned approximately 5 cm away from the stream of seeds. A plate rotation speed of ~100 rpm was used throughout for both species.

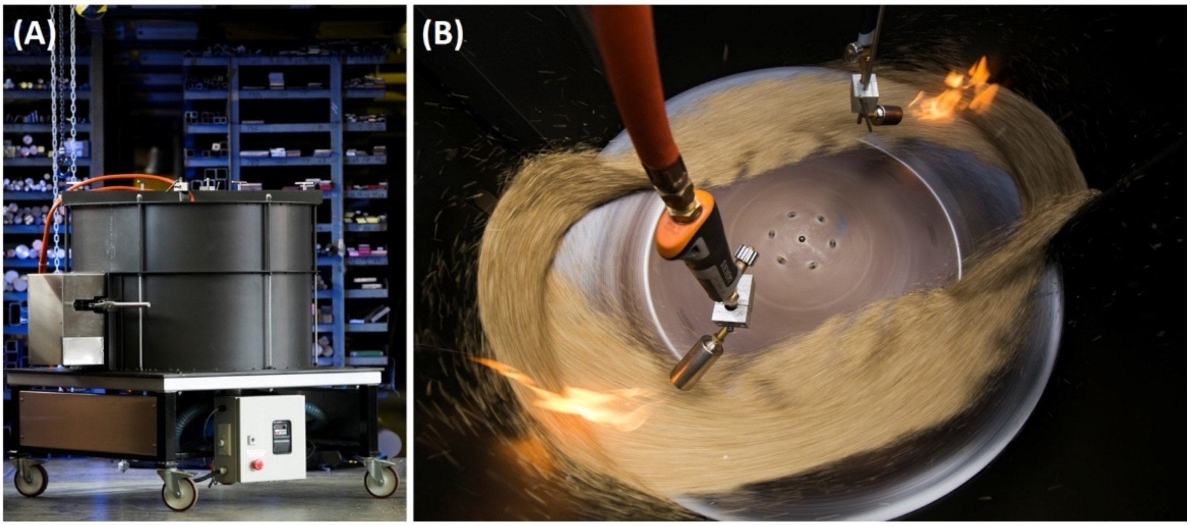

Figure S1. The flaming apparatus was supplied by the University of Western Australia and is an up-scaled (900 mm diameter) version (A) of the rotary drum coater described by Guzzomi et al. (2016) and Ling et al. (2019) with two flaming torches (B). Flame intensity, the angle and distance of the torch relative to the path of florets, and the speed of the rotary plate can all be adjusted (figure from Erickson et al. 2019).

Flowability and bulk density analysis

The weight and volume of floret material was recorded before and after flaming. Changes in these measurements were calculated to estimate improvements in bulk density (Tables S1-2).

Table S1. *Amphipogon turbinatus* volume and weight changes for each flaming treatment. The mean and standard error across all treatments is also provided.

Treatment	Start Vol (ml)	Start Weight (g)	End Vol (ml)	End Weight (g)	Vol change (ml)	Weight change (g)
SM 5L5H	1000	30	150	20	850	10
SM 10L	1000	28	150	17	850	11
LG 5L5H	1000	23	200	17	800	6
LG 10L	1000	28	250	22	750	6
**Average**	1000	27 ± 2	188 ± 24	19 ± 1	813 ± 24	8 ± 1

Table S2. *Neurachne alopecuroidea* volume and weight changes for each flaming treatment. The mean and standard error across all treatments is also provided.

Treatment	Start Vol (ml)	Start Weight (g)	End Vol (ml)	End Weight (g)	Vol change (ml)	Weight change (g)
SM 5L5H	1000	31	200	23	800	8
SM 10H	1000	32	250	25	750	7
LG 5L5H	1000	35	250	26	750	9
LG 10H	1000	34	250	25	750	9
**Average**	1000	33 ± 1	238 ± 13	25 ± 1	763 ± 13	8 ± 1

In addition to estimating the changes in bulk density, flowability testing was performed using a custom-built mechanised seeder (AUSBOX™) with a seed box funnelling down to a fluted roller which feeds seed material through up to three exit points (Figure S2). The roller has a total of 12 flutes, each of which are 8 cm^3, providing a holding capacity of 96 cm^3 per revolution. The revolution speed of the roller can be adjusted.

To accommodate the smaller experimental batches used in this study, the seed box was further divided to create a small 4 x 3 cm aperture using cardboard dividers for flowability testing (Figure S2). A speed of 10 rpm was selected for all tests. For each test a 50 ml volume of floret material was used. Each sample was placed in the small division of the seed box before the machine was turned on. The time to pass each sample was measured from when the machine was turned on to when seeds stopped falling through the exit points. Raw data of the time taken to pass each 50 ml sample is provided in Table S3.

To determine the number of florets and weight of floret material that could be passed per minute, three replicates of 5 ml samples for each treatment were weighed and the number of florets counted in each of these samples (Table S3). By combining these data with the duration (to pass 50 ml) data, the weight, volume and number of florets that could be passed per minute and the number of florets per gram were calculated for each treatment (provided in main text in Table 3).

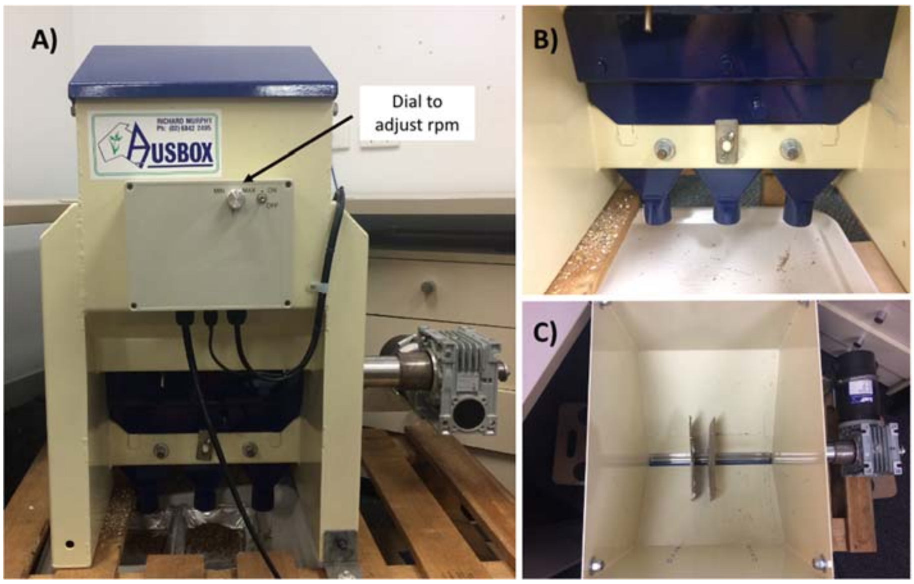

Figure S2. The custom-built mechanised seeding machine showing the entire unit and control panel (a), the three exit points (b), and the inside of the seed box with the cardboard dividers used to make a 4 x 3 cm aperture sitting directly above the fluted roller (c).

Table S3. Raw data showing the time taken (in seconds) to pass each 50 ml replicate of treated floret material for each species (*Amphipogon turbinatus* and *Neurachne alopecuroidea*) through the mechanised seeder. The mean time and standard error are provided for each treatment.

**Species**	**Treatment**	**Vol (ml)**	**R1 (s)**	**R2 (s)**	**R3 (s)**	**Mean ± SE**
A. turbinatus	Control	50	59.47	56.25	53.15	56.29 ± 1.82
	SM10L	50	35.38	30.11	30.36	31.95 ± 1.72
	SM5L5H	50	34.56	25.51	29.51	29.86 ± 2.62
	LG10L	50	23.48	26.93	28.23	26.21 ± 1.42
	LG5L5H	50	27.84	28.18	28.17	28.06 ± 0.11
N. alopecuroidea	Control	50	72	60.29	64.85	65.71 ± 3.41
	SM10H	50	17.56	18.16	19.26	18.33 ± 0.50
	SM5L5H	50	16.92	19.64	19.29	18.62 ± 0.85
	LG10H	50	20.16	18.3	17.82	18.76 ± 0.71
	LG5L5H	50	19.75	18.7	17.22	18.56 ± 0.73

Table S4. Raw data providing the weight and number of florets recorded for 5 ml samples of each treatment assessed for flowability parameters for each species (*Amphipogon turbinatus* and *Neurachne alopecuroidea*). The calculations for the mean number of florets per ml and the mean weight per ml (g) are provided with standard error. These estimates were used in calculations for all other flowability parameters (volume per minute, weight per minute, florets per minute, and florets per gram).

**Species**	**Treatment**	**Florets/5ml**			**Weight/5ml**			**Mean florets/ml (n) ± SE**	**Mean weight/ml (g) ± SE**
		**R1**	**R2**	**R3**	**R1**	**R2**	**R3**		
A. turbinatus	Control	33	42	35	0.09519	0.08797	0.07848	7 ± 0.55	0.01744 ± 0.00097
	SM10L	185	174	152	0.30687	0.31162	0.26975	34 ± 1.94	0.05922 ± 0.00265
	SM5L5H	220	245	234	0.48474	0.43555	0.41055	47 ± 1.45	0.08872 ± 0.00437
	LG10L	96	92	126	0.19370	0.17573	0.20224	21 ± 2.15	0.03811 ± 0.00156
	LG5L5H	152	136	130	0.26512	0.24561	0.24787	29 ± 1.3	0.05057 ± 0.00123
N. alopecuroidea	Control	39	31	39	0.10748	0.09428	0.11207	7 ± 0.53	0.02092 ± 0.00107
	SM10H	211	195	222	0.41693	0.42272	0.43683	42 ± 1.57	0.08510 ± 0.00118
	SM5L5H	181	172	190	0.38670	0.32793	0.38544	36 ± 1.04	0.07334 ± 0.00388
	LG10H	163	217	195	0.42762	0.44603	0.41968	38 ± 3.14	0.08622 ± 0.00156
	LG5L5H	184	168	204	0.39483	0.35418	0.43973	37 ± 2.08	0.07925 ± 0.00494

Statistical analysis and comparisons

Statistical comparisons for differences in flowability parameters including volume, weight, and number of florets passed per minute and number of florets per gram for each flaming treatment versus the control (Table S6) and all other flaming treatments (Table S7) were made. Individual t-tests were performed in Excel to allow a case-by-case definition of homogeneity of variance for each comparison. Where the assumption of homogeneity of variance was upheld a Student's t-test was used, while Welch's t-test was used where this assumption was violated. The assumption was considered violated if the standard deviation of one of the comparison groups was more than twice as large as the standard deviation of the other comparison group. There were few significant differences for *Amphipogon turbinatus* and none for *Neurachne alopecuroidea* when comparing the flowability parameters between different flaming treatments (Table S7). A reduced chaff content (rather than a true improvement in floret flowability properties) in *A. turbinatus* SM 5L5H is thought to have increased the weight per ml, leading to inflated values for weight per minute. Therefore, it was considered reasonable to pool the flaming data for the purposes of graphical representation within the main text (pooled values and statistical comparisons provided in Table S6).

For germination analysis, dose-response modelling could not be applied where germination was low or absent (< 5%) or germination rate had not decreased or plateaued. If treatments with low or absent germination needed to be compared statistically against others, the true mean maximum germination of each treatment groups was calculated directly from the raw data and compared. Because the assumptions of ANOVA were not met for these comparisons, Kruskal-Wallis non-parametric one-way ANOVA followed by Dunn's post-hoc multiple-comparisons test were used. The unadjusted p-value was used for all non-parametric post-hoc (i.e. Dunn's multiple-comparisons) statistical tests.

If maximum germination (parameter '*d*') estimates generated using the dose-response model (*drm*) were not within 5% of true mean maximum germination, parameter estimates were considered inaccurate. In these instances, germination data were recorded for a longer period to ensure estimates for parameter *d* fell within 5% of true mean maximum germination. This occurred commonly in the first round of germination testing for *N. alopecuroidea*. Therefore, germination tests for all treatments in the first round for *N. alopecuroidea* were extended to 45 d to ensure consistency in data analysis and parameter estimates.

When comparisons were made between treatments with *drm* estimates available (i.e. germination > 5%), and treatments with true mean maximum germination only (i.e. germination < 5%), the true mean was calculated for both treatments from the raw data and these compared statistically using a Kruskal-Wallis non-parametric one-way ANOVA followed by Dunn's post-hoc multiple-comparisons. T_{50} could not be calculated in these instances.

Table S5. Statistical comparisons showing the difference in mean between each treatment and the control for each species (*Amphipogon turbinatus* and *Neurachne alopecuroidea*). Comparisons are provided for variables volume (ml), weight (g) and florets (n) passed per minute, as well as florets per gram. Asterisks "*", "**", and "***" indicate significance levels 0.05, 0.01, and 0.001, respectively. Positive values indicate that the treatment performed better than the control, while negative values indicate that the treatment performed worse. The degrees of freedom (*df*) and t-values (*t*) for each comparison are provided in parentheses beneath the difference value.

Species	Treatment	Improvement on Control			
		vol/min	weight/min	florets/min	florets/g
A. turbinatus	SM 10L	41.01 ** (*df* = 3, *t* = -8.01)	0.04 ** (*df* = 2, *t* = -14.68)	2810.85 ** (*df* = 2, *t* = -19.84)	151.54 * (*df* = 3, *t* = -3.62)
	SM 5L5H	48.61 * (*df* = 2, *t* = -5.37)	0.07 ** (*df* = 2, *t* = -12.99)	4387.85 * (*df* = 2, *t* = -7.81)	105.41 (*df* = 4, *t* = -1.94)
	LG 10L	61.74 ** (*df* = 2, *t* = -9.20)	0.02 ** (*df* = 2, *t* = -11.43)	2001.62 ** (*df* = 2, *t* = -10.72)	124.03 (*df* = 4, *t* = -2.25)
	LG 5L5H	53.50 *** (*df* = 2, *t* = -29.99)	0.03 ** (*df* = 2, *t* = -28.83)	2588.13 ** (*df* = 2, *t* = -16.60)	127.14 ** (*df* = 3, *t* = -3.04)
N. alopecuroidea	SM 10H	118.04 *** (*df* = 3, *t* = -23.68)	0.06 *** (*df* = 2, *t* = -68.63)	6524.61 ** (*df* = 2, *t* = -29.14)	145.31 ** (*df* = 4, *t* = -3.62)
	SM 5L5H	115.96 ** (*df* = 2, *t* = -14.30)	0.05 ** (*df* = 2, *t* = -10.25)	5529.49 ** (*df* = 2, *t* = -16.40)	148.62 ** (*df* = 4, *t* = -1.94)
	LG 10H	114.47 ** (*df* = 3, *t* = -17.99)	0.07 ** (*df* = 2, *t* = -22.59)	5845.75 * (*df* = 2, *t* = -8.57)	97.56 (*df* = 2, *t* = -2.91)
	LG 5L5H	116.29 ** (*df* = 3, *t* = -16.83)	0.06 * (*df* = 2, *t* = -9.72)	5697.97 ** (*df* = 2, *t* = -10.50)	121.54 ** (*df* = 2, *t* = -11.74)

Table S6. Mean volume, weight, and number of florets passed per minute and number of florets per gram for the control and all flaming treatments (individual and pooled) for each species (*Amphipogon turbinatus* and *Neurachne alopecuroidea*). Standard error of the mean is provided for all values. Asterisks "*", "**", and "***" provided next to "Flamed" values indicate improvements on the control at the significance levels 0.05, 0.01, and 0.001, respectively. The degrees of freedom (*df*) and t-values (*t*) for each comparison of flaming treatment again the control are provided in parentheses beneath the mean and standard error values for each treatment..

Species	Treatment	vol/min	weight/min	florets/min	florets/g
A. turbinatus	Control	53 ± 1.73	0.93 ± 0.022	392 ± 33	423 ± 39
	SM 10L	94 ± 4.82 ** (*df* = 3, *t* = -8.01)	5.58 ± 0.003 ** (*df* = 2, *t* = -14.68)	3203 ± 138 ** (*df* = 2, *t* = -19.84)	575 ± 14 * (*df* = 3, *t* = -3.62)
	SM 5L5H	102 ± 8.89 * (*df* = 2, *t* = -5.37)	9.00 ± 0.004 ** (*df* = 2, *t* = -12.99)	4780 ± 561 * (*df* = 2, *t* = -7.81)	529 ± 38 (*df* = 4, *t* = -1.94)
	LG 10L	115 ± 6.48 ** (*df* = 2, *t* = -9.20)	4.39 ± 0.002 ** (*df* = 2, *t* = -11.43)	2394 ± 184 ** (*df* = 2, *t* = -10.72)	547 ± 39 (*df* = 4, *t* = -2.25)
	LG 5L5H	107 ± 0.43 *** (*df* = 2, *t* = -29.99)	5.41 ± 0.001 ** (*df* = 2, *t* = -28.83)	2980 ± 152 ** (*df* = 2, *t* = -16.60)	551 ± 14 ** (*df* = 3, *t* = -3.04)
	Flamed (pooled)	105 ± 3.42 *** (*df* = 13, *t* = -13.35)	6.09 ± 0.550 *** (*df* = 11, *t* = -9.39)	3339 ± 298 *** (*df* = 11, *t* = -9.84)	550± 13 ** (*df* = 2, *t* = -3.06)
N. alopecuroidea	Control	46 ± 2.34	0.96 ± 0.042	331 ± 15	347 ± 10
	SM 10H	164 ± 4.40 *** (*df* = 3, *t* = -23.68)	13.94 ± 0.001 *** (*df* = 2, *t* = -68.63)	6856 ± 223 ** (*df* = 2, *t* = -29.14)	492 ± 15 ** (*df* = 4, *t* = -3.62)
	SM 5L5H	162 ± 7.76 ** (*df* = 2, *t* = -14.30)	11.91 ± 0.004 ** (*df* = 2, *t* = -10.25)	5861 ± 337 ** (*df* = 2, *t* = -16.40)	495 ± 16 ** (*df* = 4, *t* = -1.94)
	LG 10H	160 ± 5.92 ** (*df* = 3, *t* = -17.99)	13.83 ± 0.002 ** (*df* = 2, *t* = -22.59)	6177 ± 682 * (*df* = 2, *t* = -8.57)	444 ± 32 (*df* = 2, *t* = -2.91)
	LG 5L5H	162 ± 6.50 ** (*df* = 3, *t* = -16.83)	12.89 ± 0.005 * (*df* = 2, *t* = -9.72)	6029 ± 542 ** (*df* = 2, *t* = -10.50)	468 ± 3 ** (*df* = 2, *t* = -11.74)
	Flamed (pooled)	162 ± 2.70 *** (*df* = 8, *t* = -32.51)	13.14 ± 0.445 *** (*df* = 11, *t* = -27.28)	6231 ± 234 *** (*df* = 11, *t* = -25.12)	475 ± 10 *** (*df* = 7, *t* = -8.94)

Table S7. Statistical comparisons showing the difference in mean between each flaming treatment for each species (*Amphipogon turbinatus* and *Neurachne alopecuroidea*). Comparisons are provided for variables volume (ml), weight (g) and florets (n) passed per minute, as well as florets per gram. Asterisks "*", "**", and "***" indicate significance levels 0.05, 0.01, and 0.001, respectively. Positive values indicate that the treatment listed second performed better than the treatment listed first, while negative values indicate that the treatment listed second performed worse. For example, the comparison SM 10L vs LG 10L shows that LG 10L passes a greater volume per minute, while SM 10L passes a greater weight per minute. The degrees of freedom (*df*) and t-values (*t*) for each comparison are provided in parentheses beneath the difference value.

Species	Treatment	vol/min	weight/min	florets/min	florets/g
A. turbinatus	SM 10L vs SM 5L5H	7.61 (*df* = 4, *t* = -0.75)	3.42 ** (*df* = 4, *t* = -4.91)	1577.00 (*df* = 2, *t* = -2.73)	-46.13 (*df* = 3, *t* = 1.15)
	SM 10L vs LG 10L	20.73 (*df* = 4, *t* = -2.57)	-1.19 (*df* = 4, *t* = 2.73)	-809.23 * (*df* = 4, *t* = 3.52)	-27.52 (*df* = 3, *t* = 0.67)
	SM 10L vs LG 5L5H	12.49 (*df* = 2, *t* = -2.58)	-0.17 (*df* = 3, *t* = 0.50)	-222.72 (*df* = 4, *t* = 1.08)	-24.40 (*df* = 4, *t* = 1.22)
	SM 5L5H vs LG 10L	13.12 (*df* = 4, *t* = -1.19)	-4.61 ** (*df* = 4, *t* = 6.68)	-2386.23 * (*df* = 2, *t* = 4.04)	18.61 (*df* = 4, *t* = -0.35)
	SM 5L5H vs LG 5L5H	4.88 (*df* = 2, *t* = -0.55)	-3.59 * (*df* = 2, *t* = 5.62)	-1799.72 (*df* = 2, *t* = 3.10)	21.73 (*df* = 3, *t* = -0.54)
	LG 10L vs LG 5L5H	-8.24 (*df* = 2, *t* = 1.27)	1.02 * (*df* = 4, *t* = -3.01)	586.51 (*df* = 4, *t* = -2.46)	3.12 (*df* = 3, *t* = -0.08)
N. alopecuroidea	SM 10H vs SM 5L5H	-2.08 (*df* = 4, *t* = 0.23)	-2.03 (*df* = 2, *t* = 1.88)	-995.12 (*df* = 4, *t* = 2.46)	3.31 (*df* = 4, *t* = -0.15)
	SM 10H vs LG 10H	-3.57 (*df* = 4, *t* = 0.48)	-0.11 (*df* = 2, *t* = 0.19)	-678.86 (*df* = 2, *t* = 0.95)	-47.75 (*df* = 3, *t* = 1.34)
	SM 10H vs LG 5L5H	-1.75 (*df* = 4, *t* = 0.22)	-1.05 (*df* = 2, *t* = 0.84)	-826.64 (*df* = 3, *t* = 1.41)	-23.77 (*df* = 2, *t* = 1.52)
	SM 5L5H vs LG 10H	-1.49 (*df* = 4, *t* = 0.15)	1.92 (*df* = 4, *t* = -1.59)	316.26 (*df* = 3, *t* = -0.42)	-51.062 (*df* = 4, *t* = 1.42)
	SM 5L5H vs LG 5L5H	0.32 (*df* = 4, *t* = -0.03)	0.99 (*df* = 4, *t* = -0.61)	168.48 (*df* = 4, *t* = -0.26)	-27.08 (*df* = 2, *t* = 1.63)
	LG 10H vs LG 5L5H	1.82 (*df* = 4, *t* = -0.85)	-0.93 (*df* = 3, *t* = 0.69)	-147.78 (*df* = 4, *t* = 0.17)	23.98 (*df* = 2, *t* = -0.74)

Table S8. A list of treatments for each species (*Amphipogon turbinatus* and *Neurachne alopecuroidea*) which were able and unable (i.e. producing < 5% germination) to have a dose-response model (*drm)* fitted. The round of germination testing in which treatments were included in is denoted as either (1) for round one or (2) for round two. The following abbreviations are used:
Clean = Clean seed, KAR_1 = tested on KAR_1-agar, GA_3 = Tested on GA_3-agar, SM = small flaming torch used, LG = large flaming torch used, 5L5H = 5 mins low followed by 5 mins high flaming temperature, 10L = 10 mins low flaming temperature, 10H = 10 mins high flaming temperature.

**Species**	**Treatments with *drm* fitted**	**Treatments unable to have *drm* fitted**
A. turbinatus	Clean + GA_3 (2)<br>LG 5L5H + Clean + GA_3 (2)<br>LG 10L + Clean + GA_3 (2)	Control (1)<br>Control + KAR_1 (1)<br>Clean (1)<br>Clean + KAR_1 (1)<br>SM 5L5H (1)<br>SM 10L (1)<br>LG 5L5H (1)<br>LG 10L (1)<br>SM 5L5H + KAR_1 (1)<br>SM 10L + KAR_1 (1)<br>LG 5L5H + KAR_1 (1)<br>LG 10L + KAR_1 (1)<br>Control + GA_3 (2)<br>SM 5L5H + GA_3 (2)<br>SM 10L + GA_3 (2)<br>LG 5L5H + GA_3 (2)<br>LG 10L + GA_3 (2)<br>SM 5L5H + Clean + GA_3 (2)<br>SM 10L + Clean + GA_3 (2)
N. alopecuroidea	Control (1)<br>Control + KAR_1 (1)<br>Clean (1, 2)<br>Clean + KAR_1 (1)<br>SM 5L5H (1)<br>SM 10H (1)<br>SM 5L5H + KAR_1 (1)<br>SM 10H + KAR_1 (1)<br>SM 5L5H + Clean (2)<br>SM 10H + Clean (2)	LG 5L5H (1)<br>LG 10H (1)<br>LG 5L5H + KAR_1 (1)<br>LG 10H + KAR_1 (1)

Table S9. *Amphipogon turbinatus* parameter estimates and difference between the control and each treatment. Grey shading in the first three columns indicates where it was possible to provide parameter estimates via the *drm*. In these instances, the *drm* estimate and the true mean (in brackets) are provided. Standard error is provided for all mean values.
For columns displaying change in maximum germination or T_{50m} from the control, positive and negative values indicate higher or lower maximum germination, respectively. Only changes in true mean maximum germination are provided as statistical comparisons were only possible for the true mean. Changes in T_{50m} could not be determined since T_{50m} could not be estimated for the control. Asterisks "*", "**", and "***" indicate significance levels 0.05, 0.01, and 0.001, respectively. In parentheses beneath each difference in maximum germination or T_{50m} value are the applicable test statistics. For ease of comparison, the t-value (*t*), degrees of freedom (*df*) and chi-squared (X^2) values and Z-score (*Z*) are provided.

Treatment	Maximum Germination (%)	T_{50m} (d)	Maximum germination change from control (%)	T_{50m} change from control (d)
Control	0	-	0	-
Control + KAR_1	0	-	0	-
Clean	0	-	0	-
Clean + KAR_1	0	-	0	-
SM 5L5H	0	-	0	-
SM 10L	0	-	0	-
LG 5L5H	0	-	0	-
LG 10L	0	-	0	-
SM 5L5H + KAR_1	0	-	0	-
SM 10L + KAR_1	0	-	0	-
LG 5L5H + KAR_1	0	-	0	-
LG 10L + KAR_1	0	-	0	-
Control + GA_3	0	-	0	-
SM 5L5H + GA_3	0	-	0	-
SM 10L + GA_3	0	-	0	-
LG 5L5H + GA_3	0	-	0	-
LG 10L + GA_3	0	-	0	-
Clean + GA_3	20.12 ± 4.61 (19 ± 1.12)	23.64 ± 1.95	19 * (df = 4, X^2 = 14, Z = 2.89)	-
SM 5L5H + Clean + GA_3	4 ± 0.41	-	4 (df = 4, X^2 = 14, Z = -0.94)	-
SM 10L + Clean + GA_3	0	-	0	-
LG 5L5H + Clean + GA_3	20.51 ± 1.61 (21 ± 1.12)	23.11 ± 0.61	21 * (df = 4, X^2 = 14, Z = -3.10)	-
LG 10L + Clean + GA_3	13.59 ± 2.42 (14 ± 1.19)	22.51 ± 1.61	14 (df = 4, X^2 = 14, Z = -2.19)	-

Table S10. *Amphipogon turbinatus* statistical comparisons of treatment of estimated or true mean maximum germination. Comparisons are only provided for treatments which produced germination. Grey shading indicates *drm* values were used to calculate mean difference, while no shading indicated true mean values were used to calculate mean difference. Positive values indicate the column treatment has higher maximum germination than the row treatment, and negative vales indicate the reverse. Significance levels are denoted as 'ns'= non-significant, or asterisks "*", "**", and "***" for significance levels 0.05, 0.01, and 0.001, respectively. In parentheses beneath each difference in maximum germination or T_{50m} value are the applicable test statistics. For ease of comparison, the t-value (t), degrees of freedom (df) and chi-squared (X^2) values and Z-score (Z) are provided.

**Treatment**	SM5L5H + Clean + GA_3	LG5L5H + Clean + GA_3	LG10L + Clean + GA_3
SM5L5H + Clean + GA_3	0		
LG5L5H + Clean + GA_3	-17 ns<br>(df = 4, X^2 = 14, Z = 2.16)	0	
LG 10L + Clean + GA_3	-10 ns<br>(df = 4, X^2 = 14, Z = 1.24)	7 ns<br>(t = 1.96)	0

Table S11. *Amphipogon turbinaus* statistical comparisons of treatments against their non-flamed equivalent. Grey shading indicates treatments where *drm* statistical analysis was used, while no shading indicates where true mean maximum germination was used in statistical analysis. Asterisks "*", "**", and "***" indicate significance levels 0.05, 0.01, and 0.001, respectively. In parentheses beneath each difference in maximum germination value are the applicable test statistics. T_{50m} estimates were not compared statistically so as to avoid misleading comparisons between treatments with different maximum germination. For ease of comparison, the t-value (t), degrees of freedom (df) and chi-squared (X^2) values and Z-score (Z) are provided.

**Treatment**	**Non-flamed equivalent**	**Change in maximum germination**	**Change in T_{50m}**
SM 5L5H + Clean + GA_3	Clean + GA_3	-16.12<br>(df = 4, X^2 = 14, Z = 1.94)	-
LG 5L5H + Clean + GA_3		0.39<br>(t = -0.08)	-0.53
LG 10L + Clean + GA_3		-6.17<br>(t = 1.26)	-1.13

Table S12. *Neurachne alopecuroidea* parameter estimates and difference between the control and each treatment. Grey shading in the first three columns indicates where it was possible to provide parameter estimates via the *drm*. In these instances, the *drm* estimate and the true mean (in brackets) are provided. Standard error is provided for all mean values.

For columns displaying change in maximum germination or T_{50m} from the control, positive values indicate higher maximum germination but slower T_{50m}, while negative values indicate lower maximum germination but faster T_{50m}. Asterisks "*", "**", and "***" indicate significance levels 0.05, 0.01, and 0.001, respectively. Note that T_{50m} estimates were not compared statistically so as to avoid misleading comparisons between treatments with different maximum germination. All comparisons shaded in grey use *drm* statistical analysis, while unshaded comparisons use Kruskal-Wallis and Dunn's multiple-comparisons statistical tests to derive p-values. A superscript "+" next to the treatment name indicates germination data were collected for 45 d (instead of 28 d). For ease of comparison, the t-value (*t*), degrees of freedom (*df*) and chi-squared (X^2) values and Z-score (*Z*) are provided.

Treatment	Maximum Germination (%)	T_{50m} (days)	Maximum germination change from control	T_{50m} change from control
Control$^+$	60.31 ± 2.62 (64 ± 0.71)	28.02 ± 0.77	-	-
Control + KAR$_1$$^+$	67.06 ± 2.84 (70 ± 2.53)	28.92 ± 0.79	6.75 (*t* = -1.89)	0.9
Clean$^+$ (round 1)	81.78 ± 1.33 (83 ± 0.63)	12.76 ± 0.33	21.47 *** (*t* = -7.86)	-15.26
Clean (round 2)	81.25 ± 1.67 (82 ± 0.65)	12.99 ± 0.37	20.94 *** (*t* = -7.40)	-15.03
Clean + KAR$_1$$^+$	76.14 ± 1.17 (78 ± 0.65)	11.00 ± 0.25	15.83 *** (*t* = -5.83)	-17.02
SM 5L5H$^+$	19.40 ± 2.02 (20 ± 0.91)	21.86 ± 1.97	-40.91 *** (*t* = 6.18)	-6.16
SM 10H$^+$	21.46 ± 3.53 (21 ± 1.60)	27.41 ± 3.57	-38.85 *** (*t* = 3.89)	-0.61
LG 5L5H$^+$	0	-	-64* (*df* = 13, X^2 = 50, *Z* = 3.13)	-
LG 10H$^+$	0	-	-64 * (*df* = 13, X^2 = 50, *Z* = 3.13)	-
SM 5L5H + KAR$_1$$^+$	25.30 ± 2.28 (26 ± 1.26)	26.09 ± 1.45	-35.01 *** (*t* = 5.95)	-1.93
SM 10H + KAR$_1$$^+$	17.27 ± 4.16 (17 ± 1.03)	30.41 ± 5.11	-43.04 ** (*t* = 2.92)	2.39
LG 5L5H + KAR$_1$$^+$	0	-	-64 * (*df* = 13, X^2 = 50, *Z* = 3.13)	-
LG 10H + KAR$_1$$^+$	0	-	-64 * (*df* = 13, X^2 = 50, *Z* = 3.13)	-
SM 5L5H + Clean	30.28 ± 6.43 (28 ± 2.48)	21.16 ± 2.64	-30.03 * (*t* = 2.37)	-6.86
SM 10H + Clean	18.30 ± 2.27 (18 ± 0.96)	18.09 ± 1.79	-42.01 *** (*t* = 5.44)	-9.93

Table S13. *Neurachne alopecuroidea* statistical comparisons of treatment of estimated or true mean maximum germination. Comparisons are grouped according to which subset of treatments are being compared. Comparisons for treatments including LG 5L5H and LG 10H only are not provided since germination was absent for these. Grey shading indicates *drm* values were used to calculate mean difference, while no shading indicated true mean values were used to calculate mean difference. Positive values indicate the column treatment has higher maximum germination than the row treatment, and negative vales indicate the reverse. Significance levels are denoted as ‘ns’= non-significant, or asterisks “*”, “**”, and “***” for significance levels 0.05, 0.01, and 0.001, respectively. A superscript “+” next to the treatment name indicates germination data were collected for 45 d (instead of 28 d). The t-value (t), degrees of freedom (df) and chi-squared (X^2) values and Z-score (Z) are provided.

**Flaming only**				
	SM 5L5H$^+$	SM 10H$^+$	LG 5L5H$^+$	LG 10H$^+$
SM 5L5H$^+$	0			
SM 10H$^+$	-2.06 ns<br>(t = -0.56)	0		
LG 5L5H$^+$	20 ns<br>(df = 13, X^2 = 50, Z = -1.75)	21 ns<br>(df = 13, X^2 = 50, Z = -1.68)	0	
LG 10H$^+$	20 ns<br>(df = 13, X^2 = 50, Z = -1.68)	21 ns<br>(df = 13, X^2 = 50, Z = -1.68)	0	0
**Flaming + KAR$_1$**				
	SM 5L5H + KAR$_1$$^+$	SM 10H + KAR$_1$$^+$	LG 5L5H + KAR$_1$$^+$	LG 10H + KAR$_1$$^+$
SM 5L5H + KAR$_1$$^+$	0			
SM 10H + KAR$_1$$^+$	8.03 ns<br>(t = 1.24)	0		
LG 5L5H + KAR$_1$$^+$	26 *<br>(df = 13, X^2 = 50, Z = -2.04)	17 ns<br>(df = 13, X^2 = 50, Z = -1.49)	0	
LG 10H + KAR$_1$$^+$	26 *<br>(df = 13, X^2 = 50, Z = -2.04)	17 ns<br>(df = 13, X^2 = 50, Z = -1.49)	0	0
**Flaming + Clean**				
	SM 5L5H + Clean	SM 10H + Clean		
SM 5L5H + Clean	0			
SM 10H + Clean	11.98 ns<br>(t = 1.65)	0		
**SM 5L5H only**				
	SM 5L5H	SM 5L5H + KAR$_1$$^+$	SM 5L5H + Clean	
SM 5L5H	0			
SM 5L5H + KAR$_1$$^+$	-5.9 *<br>(t = -2.27)	0		
SM 5L5H + Clean	-10.88 *<br>(t = -2.44)	-4.98 ns<br>(t = -0.87)	0	
**SM 10H only**				
	SM 10H	SM 10H + KAR$_1$$^+$	SM 10H + Clean	
SM 10H	0			
SM 10H + KAR$_1$$^+$	4.19 ns<br>(t = 0.67)	0		
SM 10H + Clean	3.16 ns<br>(t = 0.73)	-1.03 ns<br>(t = -0.22)	0	

Table S14. *Neurachne alopecuroidea* statistical comparisons of treatments against their non-flamed equivalent. Grey shading indicates treatments where *drm* statistical analysis was used, while no shading indicates where true mean maximum germination was used in statistical analysis. Asterisks "*", "**", and "***" indicate significance levels 0.05, 0.01, and 0.001, respectively. Note that T_{50m} estimates were not compared statistically so as to avoid misleading comparisons between treatments with different maximum germination. A superscript "+" next to the treatment name indicates germination data were collected for 45 d (instead of 28 d). For ease of comparison, the t-value (*t*), degrees of freedom (*df*) and chi-squared (X^2) values and Z-score (*Z*) are provided.

Treatment	Non-flamed equivalent	Change in maximum germination	Change in T_{50m}
SM 5L5H$^+$	Control$^+$	-40.91 *** (t = 6.18)	-6.16
SM 10H$^+$		-38.85 *** (t = 3.89)	-0.61
LG 5L5H$^+$		-64 * (df = 13, X^2 = 50, Z = 3.13)	-
LG 10H$^+$		-64 * (df = 13, X^2 = 50, Z = 3.13)	-
SM 5L5H + $KAR_1$$^+$	Control + $KAR_1$$^+$	-41.76 *** (t = 6.42)	-2.83
SM 10H + $KAR_1$$^+$		-49.79 ** (t = 3.04)	1.49
LG 5L5H + $KAR_1$$^+$		-70 ** (df = 13, X^2 = 50, Z = 3.47)	-
LG 10H + $KAR_1$$^+$		-70 ** (df = 13, X^2 = 50, Z = 3.47)	-
SM 5L5H + Clean	Clean	-50.97 ** (t = 3.05)	8.17
SM 10H + Clean		-62.95 *** (t = 6.37)	5.1
